# Hospice care education needs of nursing home staff in South Korea: a cross-sectional study

**DOI:** 10.1186/s12904-019-0405-x

**Published:** 2019-02-12

**Authors:** Mihyun Park, Hye-Ah Yeom, Sr Jinsun Yong

**Affiliations:** 1 The Catholic University of Korea College of Nursing, 222 Banpo-Daero, Seocho-Gu, Seoul, 06591 South Korea; 2 WHO Collaborating Centre for Training in Hospice and Palliative Care, The Research Institute for Hospice and Palliative Care, The Catholic University of Korea, Seoul, South Korea

**Keywords:** Hospices, Aged, Nursing homes, Nursing staff, Education

## Abstract

**Background:**

While the importance of hospice care education in nursing homes is recognized, the volume of research on the specific educational needs of caregivers in hospice care in nursing homes is still lacking. This study aimed to assess educational needs in hospice care among the nursing home staff in South Korea, and to examine factors related to their education needs.

**Methods:**

This is a cross-sectional descriptive study. A total of 324 nursing staff members recruited from 15 nursing homes in South Korea participated in this cross-sectional study. Measurements included demographic information, organizational characteristics, education experiences in hospice care, and educational needs in hospice care based on questionnaires developed by Whittaker and colleagues. Data were analyzed using descriptive statistics, t-test, ANOVA, and multiple regression techniques.

**Results:**

In the present study, 70.6% (*n* = 218) of respondents reported that they had previous experience with education in hospice care and expressed their continued need for further education. The provision of care in the last days of a patient’s life was the most frequent issue identified by nursing home staff for further education. Factors predicting educational needs in hospice care included provision of hospice care services in nursing homes and the existence of hospice care team meetings in the institution. Multiple regression analysis resulted in 14.3% of explained variance in the educational needs of nursing home staff in hospice care.

**Conclusions:**

Nursing home staff members showed high levels of need for training in hospice care. Therefore, it is imperative for nursing home administrators to initiate and support well-suited hospice care education for multi-level care workers on an ongoing basis.

## Background

Due to cultural influences and beliefs, the place of death is an important issue in the field of end-of-life care. Generally, most elderly people hope for dignified death in the privacy of their own home with minimal dependence on others [1]. For elderly Korean people, a peaceful death is defined as passing away at home surrounded by family and friends [2]. For nursing home residents, however, privacy needs are often difficult to meet. Because most nursing home residents share their rooms with other residents, even until the precise moment of death, their access to family and loved ones may be limited. Although variable by country, over 20% of the elderly population worldwide is known to die in nursing home [3, 4]. In Korea, the number of long-term care facilities for elderly people, including both long-term care hospitals and nursing homes, has increased more than two fold during the last 10 years [5], with approximately 2930 nursing homes currently in South Korea [6]. This phenomenon may be related to changes in social paradigms in Korea, including expanded life expectancy, an increasing number of elderly living independently, and a decreasing focus on family care giving [7].

As the number of elderly people who reside in nursing homes increases, the need for quality end-of-life care also increases. The utilization of hospice care (HC) services in nursing homes is increasing [8], and the importance of quality HC in nursing homes is widely recognized by both families and nursing staff members [9, 10]. Nursing home staff members generally have favorable attitudes toward hospice patients [11]. However, end-of-life care for nursing home residents is often a significant challenge for staff members [12], and their levels of knowledge and/or compliance with hospice and palliative guidelines are known to be low [10, 13, 14].

Hospice care aims to improve the quality of life for terminally ill patients and their families by relieving suffering and providing supportive care. Since its establishment at Calvary Hospice in 1964, hospice care (HC) in South Korea has developed remarkably in the recent past [15]. The most innovative recent change is national health insurance policies for reimbursement for HC initiated in July 2015 to improve the quality of life of patients and their families [6]. This law enables hospice patients at terminal stages to be the beneficiaries of national health insurance in community settings (home-based hospices) as well as hospital settings (hospital-based hospices or consultation-based hospices), thereby helping patients and families to receive quality HC at more affordable costs [16]. The resolution of reimbursement issues for HC contributes to the expansion and utilization of HC nationwide, and a greater number of long-term care hospitals are trying to provide HC. One criteria of HC is for caregivers to complete standard education programs as regulated by law for quality control in care systems. In the case of nursing home residents, HC is usually provided by home hospice care nurses from external hospice institutions affiliated via HC volunteers from public health centers near the nursing home [17]. While nursing homes are not directly involved in national health insurance reimbursement policies for HC services, the staff in nursing homes still need to provide hospice care as well as to collaborate with home hospice care teams for dying residents. Accordingly, the knowledge and preparedness of the nursing home staff on HC are essential.

While the importance of palliative care education in nursing homes is well recognized [18], research on targeted educational content and protocols for HC for caregivers in nursing homes are still lacking. In South Korea, the need for HC education in nursing homes is increasing [19], and the need for development of an HC protocol for nursing home staff has emerged [20]. However, specific modules and content in HC education for nursing home staffs have not been widely discussed. Education needs of various workforce groups in nursing homes should be addressed discretely, insofar as each workforce group in nursing homes including care helpers, nurses, and social workers may have different education needs regarding HC.

Care helpers, comprising the majority of nursing home staffs in South Korea, are licensed caregivers controlled by the Welfare of the Aged Act [21]. Care helpers are involved in direct basic care and in facilitating daily activities for nursing home residents. Care helpers in nursing homes differ from nurse aids in hospitals in that they are required to complete shorter periods of education for certification (240 h of education including 80 course hours, 80 h of skill training, and 80 h of clinical practice for national certification). Because nursing homes in Korea usually employ care helpers instead of nurse aids to provide direct nursing care to nursing home residents, nurse aides were not included as study participants for this study. While registered nurses play a role in supervising care helpers, social workers handle welfare-related matters for nursing home residents and provide resources to nursing home residents and their families. Thus, with various types of caregivers comprising nursing home staff, it is necessary to examine the educational needs and levels of experience in HC by work group in order to develop a systematic education protocol in accordance with advances in policy. Reflecting this gap in the literature, this study aimed to assess educational needs in HC among nursing home staff members in South Korea. Specific objectives of this study are to assess educational needs in HC among nursing home staff members, and to identify related factors.

## Methods

### Study design

This is a cross-sectional descriptive study designed to assess educational needs in HC among nursing home staff members in South Korea, and to identify related factors.

### Setting and samples

Total participants comprised 324 nursing home staffs recruited from 15 nursing homes housing more than 10 elderly patients in the metropolitan area of Seoul, South Korea. This sample size was estimated based on a significance level of .05, a power of .80, and an effect size of .15 using G-Power software. Convenience sampling was used to recruit the participants. In this study, nurse aids were not included as study participants because there were less than 10 nurse aides employed in the data collection sites and care helpers were the primary nursing workforce group who provided direct basic care to nursing home residents. Of 400 subjects initially recruited, a total of 324 staff members completed the study questionnaire, for a response rate of 81%.

### Measurements

Measurements comprised demographic information, organizational characteristics, and educational experience in HC. Demographic information included age, sex, level of education, type of work, current work position, and years of work experience at the current institution. Organizational-level information included the existence of HC services in the institution and the existence of care conferences for HC in which care providers, residents, and family members could participate. In terms of educational experience in HC, respondents were also asked about previous educational experience in HC, types of previous education in HC, intention to participate in further HC education, and preferred method of future education in HC.

Educational needs in HC were measured using questionnaires developed by Whittaker and colleagues [14] and translated into Korean by bilingual researchers, fluent in both English and Korean. Facial validity of the Korean-version scale was evaluated and confirmed by two bilingual faculty members with doctoral degrees who had no involvement in the translation process. The scale contains a total of 19 items describing HC education topics, which are consistent with the essential content of standard education programs for HC providers developed by the National Cancer Center in South Korea [22]. All items were simple in structure and straightforward in meaning; thus, the translated Korean version was considered to reflect the essential constructs of the original English version. The respondents were asked to quantify how much they needed education in each topic by selecting a score from a designated range. Each item included a score ranging from 1 (don’t need at all) to 4 (need a lot). Higher scores indicated greater educational needs in HC. The validity of the scale has been demonstrated in previous studies [23, 24]. Because the attributes of each item of the scale were heterogeneous, internal consistency reliability was not calculated.

### Data collection

Data were collected from November 2012 to March 2013. As the first step, information on nursing homes was searched on a Korean nursing home association web site (http://www.knursinghome.or.kr), which identified list of addresses of 49 nursing homes located in the Seoul metropolitan area. Of the 49 nursing homes contacted by the researchers via phone for potential participation in this study, the directors of 15 agreed to participate in the present research. Subsequently, the researchers sent survey questionnaires to participating nursing homes and collected completed questionnaires by mail in sealed envelopes. When the questionnaires were first received by nursing home administrators, they were distributed to participants by mangers or clergy persons. Each questionnaire stated that participation in the study is voluntary and that responses would be used only for research purposes. To assure confidentiality, the instructions also stated that completed survey questionnaires should be put in a sealed envelope and would not be revealed to anyone other than the researchers. To reduce redundancy, only administrators were asked for responses on organizational characteristics. Education needs were assessed by all respondents.

### Data analysis

All data were analyzed using Statistical Package for the Social Sciences (SPSS, version 18.0). As complete-case analyses showed that data were missing in random patterns, cases with missing data were not excluded from the data pool. Descriptive statistics were calculated for all study variables. Associations of general characteristics with educational needs on HC were tested using t-test and ANOVA with the Scheffé post-hoc test. Predictors of nursing home staff’s educational needs on HC were examined using multiple regression techniques. Nominal variables were dummy-coded to be entered into the regression analysis.

## Results

### General and organizational characteristics

The mean age of respondents was 50.7 years (range 19–67). Approximately 90% of respondents were women. The majority of participants were high school graduates (*n* = 201; 62%). Most of the respondents were care helpers (*n* = 242; 74.7%). The majority of total staff was involved in direct nursing care of elderly residents in the nursing homes (*n* = 207; 63.9%). The total mean length of work experience in the nursing homes was 2.3 years (range 0.1–14). Regarding organizational characteristics, over half of the respondents reported that their institutions provide HC services for residents (*n* = 202; 62.3%). However, only 26.2% of respondents (*n* = 85) reported hospice team meetings are held in the nursing homes at which they work (Table 1).

**Table 1:** General characteristics of the participants (*N* = 324)

Variables	*M* ± *SD* or *n* (%)
Age (year)	50.68 ± 9.44
Less than 35	22 (6.8)
36–45	51 (15.7)
46–55	132 (40.7)
More than 56	110 (34.0)
Gender	
Female	293 (90.4)
Male	28 (8.6)
Education	
High school	201 (62.0)
Diploma	52 (16.0)
Baccalaureate	41 (12.7)
Master’s degree	13 (4.0)
Type of work	
Care helper	242 (74.7)
Nurse	26 (8.0)
Social worker	17 (5.2)
Administrator	7 (2.2)
Other	19 (5.9)
Work Position	
Direct care	207 (63.9)
Management	38 (11.7)
Other	14 (4.3)
Work experience at the current institution (year)	2.34 ± 1.88
Less than 1	68 (21.0)
1–3	196 (60.5)
4–10	58 (17.9)
More than 11	2 (0.6)
Provision of HC service in the institution	
Yes	202 (62.3)
No	122 (37.7)
Existence of HC team meeting in the institution	
Yes	85 (26.2)
No	239 (73.8)
Previous educational experiences on HC	
Yes	91 (29.4)
No	218 (70.6)
Intention to participation in further education on HC	
Yes	264 (82.8)
No	55 (17.2)

*Note*. HC = Hospice Care. The sum of numbers in the variable was not the same with the total number because of missing data

### Educational experiences of hospice care

Educational experience in HC in each work group is presented in Table 2. In care helpers, 69.4% (*n* = 168) of respondents reported that they had previous educational experience in HC. For those with previous educational experience in HC, the most common types of education were short lectures in a 240-h comprehensive education program for care helpers (*n* = 146; 60.3%). Eighty-one percent of care helpers (*n* = 196) reported that they would like to participate in further education in HC. As methods for further education, care helpers reported preferences for video education (*n* = 109; 45.0%). In nurses, 73.1% (n = 19) reported that they had previous educational experience in HC. The most common types of previous HC education among nurses were college-based learning courses (*n* = 9; 34.6%). Over 90% (92.3%) of nurses reported needs for further education in HC. As methods for further education, care studies and group discussions were the most preferred methods of HC education in nurses (*n* = 16; 57.7%). More than half of social workers (*n* = 11; 64.7%) reported that they had previous educational experience in HC. For social workers with previous HC educational experience, the most common type of education was short lectures in a 240-h education program for care helpers (*n* = 5; 29.4%). The majority of social workers (*n* = 15; 88.2%) reported that they would like to participate in further education in HC. Social workers reported preferences for video education (*n* = 8; 47.1%) as the method for further education.

**Table 2:** Experience and Need for Hospice Care Education & Training by Type of Work (*N* = 324)

Hospice care education & training	Care helper	Nurse	Social worker
	(*N* = 242)	(*N* = 26)	(*N* = 17)
Education experience (Yes)	168 (69.4)	19 (73.1)	11 (64.7)
Education resource^+^			
University	6 (2.5)	9 (34.6)	1 (5.9)
Education program for care helpers*	146 (60.3)	2 (7.7)	5 (29.4)
Continuing education	59 (24.4)	8 (30.8)	4 (23.5)
General education program	56 (23.1)	6 (23.1)	4 (23.5)
Other	14 (5.8)	2 (7.7)	2 (11.8)
Need of further education (Yes)	196 (81.0)	24 (92.3)	15 (88.2)
Method for further education			
Lecture	93 (38.4)	7 (26.9)	3 (17.6)
Case study & discussion	105 (43.4)	15 (57.7)	7 (41.2)
Research group	13 (5.4)	3 (11.5)	2 (11.8)
Video	109 (45.0)	14 (53.8)	8 (47.1)
Visiting institutes	48 (19.8)	6 (23.1)	2 (11.8)
Others	7 (2.9)	0 (0.0)	0 (0.0)

*Note***.** ^+^Respondents could have multiple choices for the questions. * The education program is a 240 h requirement program for the care helper certification in South Korea

### Educational needs on hospice care

The mean score for educational needs in HC was 3.14. The area of greatest need in further education for HC reported by respondents was care for elderly nursing home residents in the last days of life (3.24 + 0.58), followed by spiritual care (3.22 + 0.62) and symptom management for shortness of breath (3.22 + 0.54). While the area of greatest need in further HC education among nurses was communication skills (3.56 + 0.56), the area of greatest need in further HC education for social workers was emotional support for bereaved families (3.29 + 0.59). Nurses showed higher scores than care helpers and social workers in all domains of educational needs. Specifically, among the areas of educational needs, nurses scored the highest in spiritual care (Table 3).

**Table 3:** Educational Needs on Hospice Care (*N* = 324)

Topics of education	Mean ± *SD*			
	Total	Care Helper	Nurse	Social Worker
Overview on HC				
The concept of hospice care	3.14 ± 0.53	3.12 ± 0.50	3.50 ± 0.51	3.24 ± 0.56
Goal setting in hospice care	3.09 ± 0.57	3.07 ± 0.52	3.42 ± 0.64	3.24 ± 0.56
Interdisciplinary teamwork	3.04 ± 0.58	3.01 ± 0.57	3.35 ± 0.56	3.19 ± 0.54
Pain management				
Pain assessment	3.06 ± 0.67	3.02 ± 0.67	3.48 ± 0.59	3.25 ± 0.58
Pain intervention	3.09 ± 0.65	3.06 ± 0.64	3.52 ± 0.59	3.18 ± 0.53
Symptom management				
General physical symptoms	3.11 ± 0.54	3.10 ± 0.52	3.35 ± 0.56	3.18 ± 0.53
Nausea and vomiting	3.14 ± 0.56	3.15 ± 0.52	3.42 ± 0.50	3.24 ± 0.56
Dysphasia	3.15 ± 0.53	3.15 ± 0.49	3.42 ± 0.50	3.24 ± 0.56
Constipation	3.16 ± 0.52	3.18 ± 0.50	3.38 ± 0.50	3.12 ± 0.60
Mouth problems	3.18 ± 0.55	3.19 ± 0.53	3.42 ± 0.50	3.24 ± 0.56
Dyspnea	3.22 ± 0.54	3.23 ± 0.51	3.46 ± 0.51	3.18 ± 0.64
Behavioral problem	3.16 ± 0.54	3.19 ± 0.50	3.38 ± 0.50	3.12 ± 0.60
Fatigue/frailty	3.14 ± 0.55	3.16 ± 0.51	3.44 ± 0.51	3.06 ± 0.66
Spiritual care	3.22 ± 0.62	3.21 ± 0.61	3.64 ± 0.49	3.18 ± 0.64
Others				
Nutrition/fluid balance issue	3.17 ± 0.59	3.17 ± 0.56	3.44 ± 0.65	3.18 ± 0.64
Ethical issues on HC	3.18 ± 0.60	3.20 ± 0.58	3.36 ± 0.64	3.24 ± 0.56
Communications	3.21 ± 0.57	3.23 ± 0.51	3.56 ± 0.51	3.24 ± 0.56
Care in the last days of life	3.24 ± 0.58	3.26 ± 0.56	3.56 ± 0.51	3.24 ± 0.56
Care for bereaved family	3.21 ± 0.58	3.22 ± 0.56	3.48 ± 0.59	3.29 ± 0.59

*Note*. HC = Hospice Care. The sum of numbers in the variable were not the same with the total number because of missing data

Educational needs in HC were significantly associated with level of education, type of work, and existence of hospice team meetings in the institution. Educational needs in HC were significantly higher in nursing home staff with college degrees (*F* = 3.77, *p* = .024), in registered nurses (*F* = 4.95, *p* = .002), and in those who reported no hospice team conferences within the institution (*F* = − 3.84, *p* < .001) (Table 4).

**Table 4:** Educational Needs on Hospice Care by General Characteristics (*N* = 324)

Variables	Education Needs on HC	
	*Mean* ± *SD*	*t* or *F*(*p*)
Age (year)		
Less than 35	3.15 ± 0.40	0.39(.759)
36–45	3.18 ± 0.53	
46–55	3.17 ± 0.55	
More than 56	3.11 ± 0.44	
Gender		
Female	3.15 ± 0.50	0.56(.579)
Male	3.09 ± 0.53	
Education		
High school	3.11 ± 0.51^a^	3.77(.024)
Diploma	3.14 ± 0.48	
Baccalaureate (or higher)	3.32 ± 0.46^a^	
Type of work		
Care helper	3.14 ± 0.48^a^	4.95(.002)
Nurse	3.45 ± 0.44^ab^	
Social worker	3.20 ± 0.54	
Others	2.93 ± 0.61^b^	
Work position		
Direct care	3.15 ± 0.49	2.32(.101)
Management	3.33 ± 0.57	
Others	3.08 ± 0.27	
Work experience at the current institution (year)		
Less than 1	3.14 ± 0.40	1.35(.260)
1–3	3.11 ± 0.53	
Over 4	3.24 ± 0.50	
Provision of HC service in the institution		
Yes	3.12 ± 0.49	−1.30(.196)
No	3.19 ± 0.51	
Existence of HC team meeting in the institution		
Yes	2.97 ± 0.46	−3.84(.000)
No	3.20 ± 0.50	
Total	3.14 ± 0.50	

*Note*. HC = Hospice Care

Multiple regression analyses with a simultaneous approach were conducted to examine factors influencing educational needs in HC. General characteristics and previous educational experience in HC were entered in the regression model as predictors of educational needs in HC. Assumption of the multi-collinearity of variables was met insofar as the variance inflation factor (VIF) values were not larger than 10. In addition, linearity, normality, and equal variance of the equation model were supported through residual analysis. Factors significantly predicting educational needs in HC included the provision of HC services in nursing homes (*ß* = −.19) and the existence of HC team meetings in the institutions (*ß* = −.19). Multiple regression analysis explained 14.3% of the variance in educational needs in HC (Table 5).

**Table 5:** Factors affecting Educational Needs on Hospice Care (*N* = 324)

Variables	B	S.E.	*ß*	t	Sig.	R^2^	F(*p*)
Age	−.002	.004	−.033	−.489	.625	.143	2.508 (.001)
Gender^†^ (1 = female)	.068	.106	.039	.646	.519		
Education^†^ (1 = High school)	.067	.092	.050	.729	.467		
Type of work^†^ (1 = Administrator)	.288	.208	.089	1.386	.167		
Work position^†^ (1 = Management)	.032	.114	.019	.278	.781		
Work experience	.014	.016	.051	.832	.406		
Provision of HC service in the institution (1 = Yes)^†^	−.673	.276	−.193	−2.44	.015		
Existence of HC team meeting in the institution^†^ (1 = Yes)	−.175	.079	−.155	−2.22	.027		
Previous education experience on HC participate in^†^ (1 = Yes)	−.076	.065	−.071	−1.16	.246		

^†^ Dummy coded

## Discussion

Most respondents (70.6%) in the present study reported that they had previous education in HC and expressed their continued need for further education. This need for education in HC in nursing home staff members is consistent with the findings of previous research conducted in nursing home staff [14], suggesting that education is a core component in the provision of quality HC in a nursing home setting. Because most types of HC education reported by nursing home staff members were one-time lectures, which did not provide comprehensive or practical information on HC, staff members have expressed the need for more in-depth HC education tailored for a nursing home setting.

The majority of nurses (73.1%) were shown to have previously experienced HC education, which is similar to the findings of previous research wherein over two-thirds of nurses working in geriatric hospitals were trained in HC [25]. These results indicate that nurses are fairly well prepared for end-of-life care in care settings for geriatric patients in Korea.

The level of educational needs in HC in this study was 3.14 out of 4 points, showing a high level of need among nursing home staff members for ongoing training in HC. In the present study, most participants were care helpers (74.7%) with a high school degree and who were involved in the direct care of elderly residents by assisting with activities of daily living. Care helpers in this study showed the strongest need for education in areas of care in the last days of life and communication with nursing home residents. It is widely agreed that strategies for communication between staff and patients and/or their families at end-of-life stages in nursing home settings are important [26, 27]. Regardless, sufficient protocol or guidelines for effective communication strategies in HC are scarce for care helpers in Korea. The recommendation is that HC educational guidelines should be easy to understand, specific to the learning needs of care helpers, and sufficiently practical for implementation in the daily routines of care helpers. Development of user-friendly manuals on care for dying residents and communication strategies for care helpers is needed in the future.

By workforce group, registered nurses showed higher levels of educational needs in the domains of pain management and spiritual care, suggesting that registered nurses require ample education in these areas. In comparison to hospital settings where the majority of nursing staff are registered nurses, nursing home staffs tend to be care helpers who are non-healthcare professionals with limited formal nursing education. For the area of greatest need in HC education among social workers, care for bereaved families was the most challenging issue reported. This implies that social workers in nursing home settings in Korea encounter challenges in caring for families who experience the loss of loved ones and thus need ongoing education in care strategies for bereaved families.

Whereas hospice patients in hospital settings are most likely to pass away from terminal cancer, the majority of nursing home residents die from complications due to other chronic illnesses, including dementia, pneumonia, and health failure [28]. The deaths of nursing home residents are often unpredictable, and signs and symptoms of impending death are not well recognized by nursing home staffs [29]. This unpredictable nature of death in nursing home residents contributes to ambiguity in knowing the accurate signs and timing of death in residents, thus imposing challenges on caregivers to provide appropriate support for residents in the last days of their lives. Therefore, further education in HC for nursing home staff in Korea should focus on palliative care-oriented approaches, including incorporation of the philosophical basis of HC by nursing staffs for everyday caregiving practices on behalf of nursing home residents. Specifically, HC education should emphasize the importance of chronic illness management and the improvement of specific care skills for symptom management in residents with imminent death.

In the prediction model, organizational characteristics including the provision of HC services and the existence of care conferences in HC for residents within institutions were found to be factors influencing educational needs in HC among nursing home staff. In the present study, over half of the participating institutions provided HC services for residents, but only one-third of participating institutions held regular care conferences in HC for staff. It is well recognized that the participation of family members in interdisciplinary care conferences has positive impacts on effective communication and organizational identity for caregiving staff [30, 31]. It is plausible that care conferences in HC may increase exposure of staff members to communication strategies for end-of-life care issues with hospice patients, thereby improving the knowledge and attitudes of staff members toward HC. Accordingly, organizational support for various types of education in HC may motivate nursing home staff to learn and implement HC into their daily care routines of residents. Unique HC education needs of heterogeneous workforce groups in nursing homes should also be recognized and understood from the standpoint of management in order to design and implement tailored HC education programs for each work group, which ultimately stands to improve quality in end-of-life care of the institution.

There are several limitations of this study. The cross-sectional study design limits the proposition of causal relationships among variables. The current study was conducted at nursing homes located in the Seoul metropolitan area. Therefore, results may not be generalizable to nursing homes in other geographic regions of South Korea**.** In this study, only forward translation was done for the Korean-version educational need assessment scale without reverse translation of the scale. Therefore, construct validity of the Korean-version scale should be further tested in future psychometric studies. The tested set of variables accounted for only 14% of observed variance in educational needs in HC, suggesting that a structural equation modeling approach may help determine latent factors to explain more variance in the HC educational needs of nursing home staff members in future studies. Nevertheless, the findings of this study contribute to the existing body of knowledge on HC by describing HC educational needs of nursing home staff in Korea. Further research should focus on developing nursing home specific education protocols for nursing home staff with contents to fit end-of-life care for nursing home residents in Korea.

## Conclusion

In the present study, nursing home staff showed high levels of need for further education in hospice care. By workforce group, frequently reported issues in need of further education were care in residents’ last days of life among care helpers, spiritual care of residents among nurses, and care for the bereaved families of residents among social workers. Factors significantly predicting educational needs in HC included the provision of hospice care services in nursing homes and the existence of interdisciplinary staff meetings in the institutions, which are related to system modifications.

## Abbreviation

HC: Hospice care
